# Traffic prediction in SDN for explainable QoS using deep learning approach

**DOI:** 10.1038/s41598-023-46471-8

**Published:** 2023-11-23

**Authors:** Getahun Wassie, Jianguo Ding, Yihenew Wondie

**Affiliations:** 1 IP Networking and Mobile Internet, Addis Ababa University, Addis Ababa, Ethiopia; 2 Department of Computer Science, Blekinge Institute of Technology (BTH), 371 79 Karlskrona, Sweden; 3 Department of Electrical and Computer Engineering, Addis Ababa University, Addis Ababa, Ethiopia

**Keywords:** Computer science, Information technology

## Abstract

The radical increase of multimedia applications such as voice over Internet protocol (VOIP), image processing, and video-based applications require better quality of service (QoS). Therefore, traffic Predicting and explaining the prediction models is essential. However, elephant flows from those applications still needs to be improved to satisfy Internet users. Elephant flows lead to network congestion, resulting in packet loss, delay and inadequate QoS delivery. Recently, deep learning models become a good alternative for real-time traffic management. This research aims to design a traffic predicting model that can identify elephant flows to prevent network congestion in advance. Thus, we are motivated to develop elephant flow prediction models and explain those models explicitly for network administrators’ use in the SDN network. H2O, Deep Autoencoder, and autoML predicting algorithms, including XGBoost, GBM and GDF, were employed to develop the proposed model. The performance of Elephant flow prediction models scored 99.97%, 99.99%, and 100% in validation accuracy of under construction error of 0.0003952, 0.001697, and 0.00000408 using XGBoost, GBM, and GDF algorithms respectively. The models were also explicitly explained using Explainable Artificial Intelligence. Accordingly, packet size and byte size attributes need much attention to detect elephant flows.

## Introduction

Today, the Internet holds traffic from various real-time applications with different QoS requirements. The existence of new and varied multimedia applications generates elephant flows (large flow size) which require a higher data loss rate, more considerable bandwidth, and a longer delay^1^. These significant flows create congestion problems due to the unfair use of resources in switches, routers and controllers, degrading network performance dissatisfying Internet users^2^.

QoS is defined as the ability of Internet service providers (ISPs) to deploy network infrastructures and devices to support a certain level of assurance to a specific Internet service, enhancing performance and reliable data delivery^3^. Network traffic is considered an elephant when data volume and duration exceed certain classification thresholds^4^. Hence, QoS imposes requirements from service providers such as detecting jams, visualizing and explaining QoS provision before congestion happens. Historically, QoS improvement relies on TCP or UDP port matching. Still, more than the port-based techniques such as deep packet inspection (DPI), is needed for traffic management from thousands of fabricated applications. To fill the limitation of DPI, the state of the art deep learning algorithms(ML) have been preferred to detect, classify, cluster and track elephant traffic of the applications^4^. Deep learning predicting algorithms are more promising for traffic management of elephant flows in this considerable data age^5^.

Before building predictive models, clustering algorithm, H2O, was used to cluster the unlabeled traffic data. Clustering focuses on discovering patterns from unlabeled flows with similar characteristics that are grouped into clusters without prior guidance from human class labeling. Thus, clustering can potentially learn class label automatically from multimedia traffics pattern^6^.

H2O is used to automatically label traffic flows as elephant and mice flows. H2O’s deep learning functionalities include distributed parallel computation that can be run on a single or multi-node cluster^7^.

After class assignment automatically, the next task is to remove anomalies from the SDN dataset to generate a more reliable elephant predicting model. These Anomalies data were removed using a deep Autoencoder algorithm. Deep Autoencoder transforms inputs into outputs with the least possible distortion (construction errors). Auto-encoders are integrated and play a crucial role in deep Autoencoder to remove exceptional data (anomalies). Mainly, Autoencoder plays a fundamental role in unsupervised learning extensively used to set a threshold boundary to marginalize network traffic used threshold value.

Threshold improves the quality of predicting models in terms of accuracy^8^. 0.091 was the threshold value obtained as an anomaly traffic boundary. The anomaly data greater than this threshold are removed to provide healthy labeled dataset and maximize autoML predicting model performance. Once the predicting model is developed, every data instance including healthy or anomaly is treated as elephant or mice.

The built model is explained further for more description of the model. The black box constraint is solved using the Explainable Artificial Intelligence(XAI) mechanism to show attribute importance and effect on the predicting model^9^^,^^10^. Moreover, we used SHAP to explain the model interpretability.

The proposed Predicting model can be fully integrated into the network controller or switch for real-time, adaptive, and accurate elephant flows prediction task. It predicts elephant’s flows and visualizes and controls the most influential feature during traffic transmission. Predicting features facilitates network service providers to make various network management decisions such as network maintenance, network optimization, and routing policy setting, load balancing, protocol design, anomaly detection and prediction of future traffic trends^11^.

## Motivation

Multimedia data from a real time application need further attention to solve the unsolved, aged and practical problem of traffic congestion^12^. Multimedia applications, including video streaming, and VoIP generate elephant flows that require high bandwidth and low latency.

Therefore, the global network character of SDN is one of the network models which minimize the network congestion problem due to the central controller. For this purpose, QoS is monitored in SDN using openflow protocol. However, Openflow Protocol requires additional components to predict the presence of elephant flows in advance, which in turn helps to assign optimal routes for the traffic. Furthermore, network traffic prediction can predict future traffic by learning from historical data, which serves as a proactive method for network resource planning, allocation, and management in SDN architecture^13^.

Hence, we are motivated to add value to SDN by integrating deep learning based traffic predictors. The work can assist network administrators in reserving QoS and predicting appropriate routes automatically. Predicting traffic size helps to reserve routes automatically based on the traffic load/capacity of each link in advance, making traffic administration easier and more satisfactory in service Providers and QoS consumers.

## Related work

Recent literatures have focused the ever-increasing number of real-time applications and their traffic from Internet-of-Things (IoT)^14^, Internet of vehicles (IoV)^15^, cellular communication (5G)^16^, multimedia network^17^, wireless mesh networks^18^,SDN networks^19^. Specifically, delay and bandwidth sensitivity of real time and multimedia applications such as VOIP and Video conferencing have been studied for elephant flows management. For instance, QoS of VOIP for different broadband networks was evaluated using different QoS parameters such as end-to-end delay, throughput and jitter in paper^20^. Therefore, identifying and handling elephant flows is a proactive task for QoS provisioning. In this regard, networks such as vehicle network(VNs) requires a proactive warning of traffic jam mechanism to prevent congestion in advance^21^. To make flow decisions based on flow size, a global view of the network topology is feasible. For this reason, the SDN network is a potential option to control heavy hit traffic, elephants. Many authors^19^ researched SDN networks to detect elephant flows.

In the paper^19^, the authors optimized elephant flow management in SDN networks using deep learning algorithms. Deep learning has recently gained high trust for solving network congestion problems in SDN. However, the elephant detection model requires further visualization and explanation for network experts about the probability of jams in the network in advance. Hence, the study of QoS provisioning using SDN and deep learning technology gained strength in this context^13^. Furthermore, elephant flow prediction in SDN needs a fine-grained and descriptive manner for improved QoS^2^.

Author^22^ used the sFlow technique in SDN to detect elephant flow. The separation of control and data planes, global centralized control, and programmability of network behavior help more to identify both TCP and UDP elephant flows. Elephant flows can be classified into large and long-lived flows, whereas mice flows are small and short-lived flows^2^.

Unlike SDN global traffic management, authors^18^ employed a federated learning technique to train their models locally and only periodically exchange models with a central server. This approach enables the network to reduce reliance on the server, reduce high communication costs, respect the privacy, and potentially improve robustness. Furthermore, the author^23^ enhanced their works by introducing an automatic data collector module to minimize communication and processing costs.

However, elephant flow detection for optimizing QoS needs a traffic overload prediction mechanism.

In the paper^24^, traffic predicting techniques are used to predict the probability of traffic jam in 5G networks. Predicting traffic was made in the context of different types of applications. These different types of applications have different levels of QoS requirements. Thus, elephant flow prediction becomes critical due to SDN`s global view and programmable advantage. In their work, predicting models were unhidden while model visualization generated and generate illustrations of the predicting model’s internal operations and interaction. For this shortcoming, author^10^ XAI makes predicting models interpretable, manageable, and trustworthy in practice. In addition to this, the XAI application explains deep learning model. SHAP explains the specific predicting results and detects the most influential features representing elephant flows. This in turn, reduces the most determinant attribute and feature to control elephant flows during network transmissions. Moreover, network administrators control traffic congestion using visual heuristics.

Inspired by the previous works, we developed a traffic flow prediction model and explained it using the XAI tool, SHAP. As a result, ISPs can provide good QoS in a negotiation manner with users accessing good QoE. We construct a predicting model using SDN dataset and demonstrated how explanations can be used to monitor traffic congestion and provide QoS decisions as well as aid in the visualizing of potential features which cause traffic congestion. To conclude, we summarized the most related works in Table 1.

**Table 1:** Summary of literature on traffic prediction.

Reference	Author	Year	Traffic dataset	Approach	Experimental results	Key contributions				
						SDN	ML/DL	XAI	QoS	Elephant flow
^2^	Muna Al-Saadi and et al	2023	Generate data from the network	Machine learning	High throughput, and data transfer rate(88.2% accuracy)	✓	✓	x	✓	✓
^18^	Cheng Qiao, Zhihong Tian, Kenneth N. Brown, Fan Zhang	2023	Generated dataset from network	Federated learning	Reduce high communication costs and respect the privacy and reduce failures	X	✓	x	✓	x
^21^	Chen, Zhiwen, et al	**2023**	Generated data from the network	Distributed algorithm (K-mean) for VNs	Declined network overhead(53%), and improve accuracy	X	✓	x	✓	x
^19^	Ali Malik et al.	2020	Moore datasets	Deep learning	Predicting model with promising average accuracy (0.936%)	✓	✓	x	✓	✓
^23^	Rahma Gantassi,et al	2021	Generate data from the network	Machine learning	Improves energy consumption and QoS	✓	✓	x	✓	x
^4^	Silva, Marcus Vinicius Brito da, et al.	2020	Data generated using sFlow	sFlow	Identify and react to elephant flows quickly(< 0.4 ms) and achieved 90% accuracy	✓	✓	x	✓	✓
^10^	Pieter Barnard, et al	2020	Networking data	Machine learning then XAI(SHAP)	Explain the the predicting model and identify important features	X	✓	✓	✓	x
Our paper	Getahun W, Jianguo Ding, Yihenew W			Deep learning	Predict, visualize, and explain elephant flows in advance with promising accuracy	✓	✓	✓	✓	✓

## Methodology

### Traffic predicting model in SDN for good QoS

In provisioning QoS for real-time traffic, the proposed QoS provision in SDN improves users` QoE to get appropriate QoS requirements on demand^25^. To address this objective, H2O deep learning-based framework is employed to optimize the network performance by labeling classes’ automatically.

The QoS optimization has three parts. Firstly, H2O categorizes elephant and mice flow automatically. This part was responsible for partitioning the traffic into distinct groups based on performance metrics such as packet loss, round trip time (RTT), and throughput by using an unsupervised algorithm, H_2_O^7^. Secondly, the Deep Autoencoder creates a threshold value to remove anomalies above this threshold^4^. Thirdly, the predicting model predicts the probability of elephant flows in a more explained manner using SHAP^26^. The effect of each feature is quantified and presented based on real-time application behavior, and threshold values for the predefined parameters such as a number of packets, flow size, application protocol and duration as heuristics^27^. Attribute and feature importance are presented for making a dynamic model to enhance QoS^26^.

### Architecture

Several factors motivated the rise of approaches that attempt to turn predictive black-boxes transparent to the decision-makers. We are motivated to explain the elephant flow and QoS requirements.

While elephant flow prediction development, procedures starting inputting dataset to detecting elephants and visualizes the influencing features.

**Figure 1:**
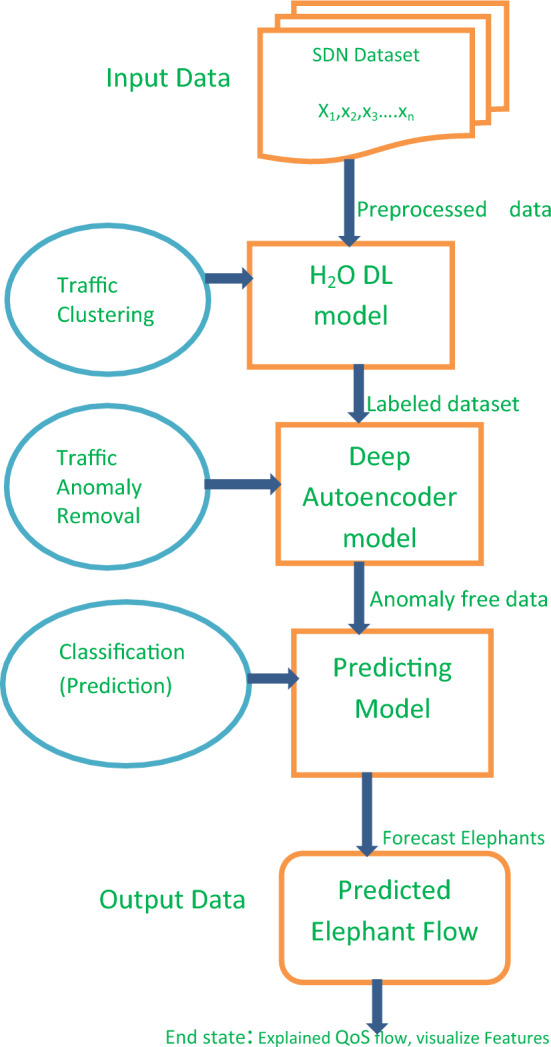
Traffic Predicting Architecture.

Clustering for label assignment, abnormal data removal and developing the prediction models are the core tasks to explain the model for its explicit visualization. Predicting the probability of the occurrence of elephant flows given the previous train set, is prerequisite to identify the main factors for congestion. This concept is presented in Fig. 1.

#### Input dataset

This is an SDN specific data set generated by using a mininet emulator in which switches are connected to a single ryu controller. It is used for traffic classification by machine learning and deep learning algorithms. The dataset is used to simulate and detect elephant flows from TCP, UDP and ICMP traffic. A total of 23 attributes are available in the data set in which some are extracted from the switches and others are calculated^28^. These attributes are presented in Table 2.

**Table 2:** Attributes of SDN dataset.

Extracted features	
Switch	Switch-id
pktcount	Packet count
byte_count	Byte count
dur	Duration in seconds
duration_nsec	Duration in nano seconds
tot_dur	The total duration of packets
src	Source IP
dst	Destination IP
port_no	Port number
tx_bytes	Bytes sent by functions
rx_bytes	Bytes received by functions
dt	Field shows the date and time which has been converted into number and a flow is monitored at a monitoring interval of 30 s
Flows	Flow duration
pktrate	Rates at which packets arrive
Protocol	Protocols (TCP, UDP, ICMP)
Calculated features	
pktperflow	Counts of packets in flows
byteperflow	Byte per flow is the byte count during a single flow
pktperflow	The packet rate is number of packets send per second and is calculated by dividing the packet per flow by monitoring the interval
packetins	Input packets from devices
Pairflow	Flows in pairs
tx_kbps	Transmission from sender to receiver in kilobit per second
rx_kbps	rx_kbps are data transfer and receiving rate and Port Bandwidth is the sum of tx_kbps and rx_kbps
tot_kbps	Tolal kilo bit per second
Label	Elephant (1) or mice (0) flows

The dataset contains 104,345 records and 23 attributes, x_1_, x_2_, x_3_….x_21_. We modified the datasets class labels values in line with QoS requirements such as packet size and flow duration. Specifically, Elephant flows take at least 10 s of flow duration^29^ and hold at least 15 packets, each packet with 500 bytes^30^. Having this heuristic information, traffic labels have elephant or mice class values. Elephant flow has label 1 whereas mice traffic has label 0. After preparing the dataset in such a way, this SDN dataset is automatically split into a training set and a test set in a cross-fold validation method, starting with fold 1 and ending with fold 5 using fivefold cross-validation.

#### H_2_O clustering

H2O is a fast, scalable, open algorithm with advanced algorithms such as deep learning boosting algorithms^31^. H2O deep learning has been used for better traffic clustering in elephant and mice category. The H2O clustering task is required to evaluate how much its performance is near to the supervised class labeling task. We pretend that we don't have the labeled traffic although we train on mice and elephants supervisory. But, we use labels of instances is generated from H2O clustering task.

In practice, flow length and size distributions are even more long-tailed than the Pareto rule (80/20) assumes. According to a recent analysis, 80% of traffic is caused by only 20–40% of flows^32^. Hence, it is true that our 35% SDN records are categorized under the elephant flow cluster which is in between 20 and 40% of flows. So, we can entail that elephant flow management facilitates QoS provisioning in the SDN network.

#### Deep learning AutoEncoder

The Autoencoder is a kind of unsupervised learning which can be used as a feature extractor of data in order to reduce dimensions or extract features. The weight matrix after training retains the original data`s characteristics before training the weight matrix.

If the extracted feature can reconstruct the original data well, it indicates that the features of the original data can be effectively retained through the weight matrix^33^.

A dynamic threshold mechanism is needed in order to detect reconstructed errors statically data. Unlike standard deep learning techniques which learn the neural network weights for classification, Auto-encoders do not require any human label except the label obtained automatically using H2O. It learns from data with instances with their label to reconstruct the input to get the threshold^34^. The Autoencoder model has 16, 8, 4, 8, and 16 hidden layers structure.

Before prediction, the deep Autoencoder model helps to remove anomaly data and train the traffic model upon the normal traffic dataset by setting the batch size to 256, epochs 10 using fivefold cross-validation. This activity directly contributes to optimize QoS since elephant flow prediction can provide heuristics about the traffic load conditions in advance.

#### Supervised model prediction

eXtreme gradient boosting (XGBoost) is a gradient boosting extension that is reliable and efficient machine learning algorithm^35^. Gradient boosting machine (GBMs) does not require a priori knowledge about the data structure, such as a class label. GBM is used to discover the most important variables and their relationships for traffic prediction tasks^36^. However, gradient boosting algorithms are known for black-box effects, i.e. it can be difficult to interpret how the models work. Hence, we used the XAI technique, called SHAP for our GBM model interpretation. We also employed the Deep Random Forest (DRF) algorithm; one of the most successful machine learning algorithms composed of decision trees generated using randomization. Random Forests are an ensemble of regression trees based on bagging.

##### Predicting model evaluation

The proposed model was evaluated using accuracy and precision. An elephant predicting result has four cases: True Positive (TP), False Positive (FP), True Negative (TN), and False Negative (FN). Accuracy is the performance metric that accounts for these four cases. So accuracy is calculated using the formula:

1 $$\mathrm{Accuracy}=100*\left(\begin{array}{c}\frac{\mathrm{TP}+\mathrm{TN}}{\mathrm{ TP}+\mathrm{FP}+\mathrm{FN}+\mathrm{TN}}\end{array}\right),$$

2 $$\mathrm{Precision}=100*\left(\begin{array}{c}\frac{\mathrm{TP}+\mathrm{TN}}{\mathrm{ TP}+\mathrm{FP}+\mathrm{FN}+\mathrm{TN}}\end{array}\right).$$

In addition to accuracy and precision, we use the loss function.

#### Explain QoS using XAI

To cope with these blackbox limitations, XAI namely, SHapley Additive exPlanations (SHAP) has been used to interpret the behavior of a state-of-the-art traffic classifier and predicting models^23^. We choose SHAP because it assigns values to features in a prediction and enables us to identify the driving factors(elephants) behind traffic congestion and network administrators` decision, making the elephant prediction model more interpretable and trustworthy^10^. Interpretability is about extracting relevant sub-symbolic information from a built deep learning model concerning relationships either contained in data or learned by the model^37^. Furthermore, model explain ability is translating this sub-symbolic information comprehensibly through human-understandable language expressions using statistical quantities and visualizations. Explaining the model helps, network administrators and ISP operators to control the traffic based on features or attributes. The negative impact of each feature can be visualized. In this regard, SHAP visualizes the backbox model with quantifiable and visible traffic values. Thus, it helps to know the model`s content explicitly.

XAI techniques are used to identify the top Causality of traffic congestion. Causality is about explaining a statement to a human expert to have a specified level of causal understanding with effectiveness, efficiency, and satisfaction in a specified QoS need^37^.

The notion of causality within this study is to predict and identify the leading cause of elephant flows which causes traffic congestion. It creates interpretable and explainable methods that explain to the network administrators why certain features contributed to a specific elephant flow prediction^37^. Doing so, helps the administrators allocate optimal routes to achieve a better QoS and maximize QoE for Internet users. For this sake, SDN-based attribute and feature importance are investigated using XAI. Specifically, SHAP is used to identify the main factor that brings Elephant flows. Identifying the main features which lead to network congestion is critical task for QoS optimization.

### Ethics approval and consent

We take care about plagiarism and we do know to have human-contacted experiments except the cited dataset used for our paper.

## Experimentation and evaluation

This study aims to build a model that best predicts elephant flows to prevent traffic congestion in advance. Elephant flow accounts for 35% of the datasets whereas 65% of the traffic dataset is mice flows using the H2O clustering algorithm. Here, we do not use the model itself for Elephant flow prediction, rather we used it to label instances automatically instead of label assignments using supervised class annotators.

We got 65% of the records are mice flows. The rest, 35% of the records are elephant flows. This imbalance is not a problem for our model. The main point is how much the clustering model performs compared to the manual label assignment. We got that the manual and the clustering approaches perform almost the same. Therefore, we opt for labels from the automatic clustering process. Then, we eliminate any anomalies from the SDN dataset for developing a predicting model. We used deep autoencoder to get the threshold value to remove anomalies that can mislead the prediction process. To meet this goal, features from the SDN dataset were extracted for training and testing using deep autoencoder algorithm.

After identifying elephant and mice labels for each instance using H_2_0 and removing anomalies using deep Autoencoder, we employed autoML algorithms (XGBoost, GBM, and GDF) to develop prediction models. Predicting elephant flows using gradient boosting algorithms is helpful in predicting causes of traffic congestion in advance. It also provides insights to network administrators to deliver good QoS and optimal routes.

This chapter discusses the experiment setups, model building, experimental results, and its result discussions. We conduct experiments following the research questions.

### Experimental setup

Deep learning components were used to implement the traffic predicting experimentation on Notebook Editor. Some components are numpy, pandas, matplotlib, and SHAP framework on top of TensorFlow. Python 3.9.16 under the UNIX operating system using 13.62 GB RAM from the colab environment was used; the detailed hardware and software tools are:

- Python Version: 3.9.16
- Processor type: x86_64, Intel(R) Xeon(R) CPU @ 2.20 GHz
- Operating system: Linux, release: 5.10.147 + 
- Total RAM installed: 13.62 GB.

### Discussion of experimental results

*Experiment I*: How much do the H_2_O clustering model, Deep Autoencoder and predicting models perform for predicting elephant flows?

#### Evaluation of the H_2_O cluster model

To evaluate the clustering model performance, 40,784 records were assigned Elephant class and 63,561 records were assigned to the mice class label in a supervised manner. According to this assignment, 39.08% of instances from the total dataset were grouped in the elephant class manually. The manual supervised class assignment was a reference for the H_2_O-based clustering model. The groupings of these records into elephant flows were checked automatically using H_2_O clustering algorithm. Accordingly, 39.11% of records were clustered in the elephant category using the H_2_O algorithm. Therefore, we can conclude that instances with elephant labels are almost equal in both manual count and H_2_O cluster results.

Therefore, we can conclude that training and testing elephant splits ensure the elephant flows equal distribution which assures the performance of the clustering model has the potential to categorize elephant and mice flows automatically. Therefore, instances with their associated automatic labels were used for predicting model development.

After performing this checkup, we developed a deep Autoencoder model to remove construction errors using thresholds mechanism. Deep Autoencoder eliminates anomaly data such as missing data or any other exceptional instances of features from the dataset. 20 columns with different categorical, integer and real number features(x) and 1 target column (y) were preprocessed using H_2_O and deep Autoencoder. The target variable (class) is binary which can take values "yes" if the traffic is elephant or "no" if the traffic is mice.

#### Evaluation of the DNN auto-encoder model

The DNN auto-encoder model training uses a dense layer after data preparation. Specifically, the H_2_ODeepLearningEstimator of Deep Learning is used to develop the autoencoder model. The Reported MSE of train data becomes 0.043. The ModelMetricsAutoEncoder of deep learning reported 0.042 MSE on validation data.

**Figure 2:**
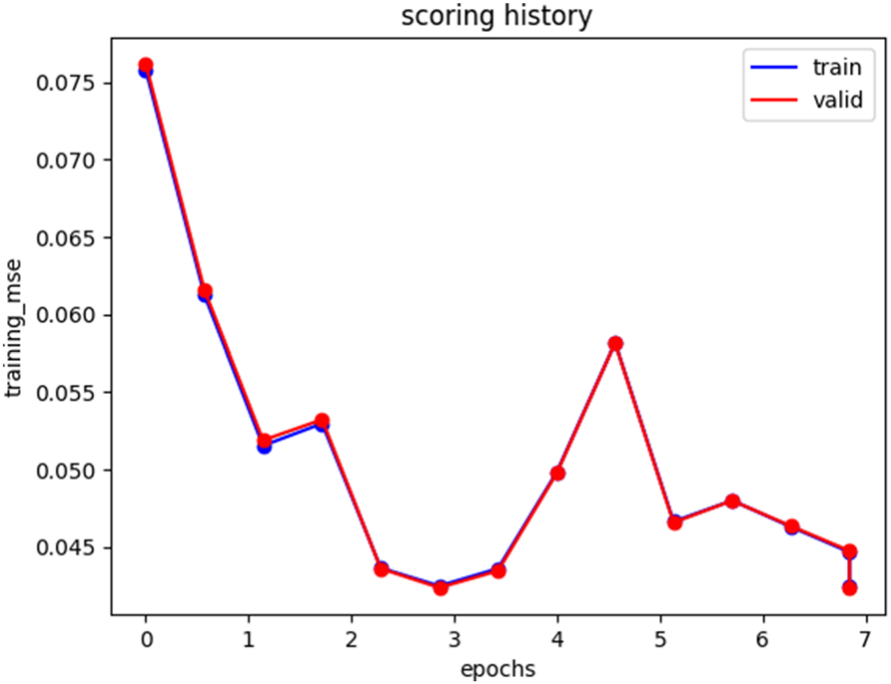
DNN Autoencoder model training history.

If the MSE error is closer to zero, it assures that the traffic model is promising performance. The model reconstruction loss decreases smoothly and approaches zero as its training history is seen in Fig. 2. Specifically train data loss decreases from 0.078 to 0.043 in MSE and validation data loss decreases from 0.076 to 0.042 in MSE. Reconstruction MSE error is a threshold separator between error and regular traffic (elephant and mice flow). The threshold was 0.091 which separates traffic flows with error (anomalies). Data instances that are greater than the threshold value are eliminated from the dataset. They are assumed to be errors and they are removed from the dataset. This action is more beneficial to build a prediction model from healthy traffic.

The training and validation reconstruction error almost overlaps due to minimum over-fit and under-fit constraints.

**Figure 3:**
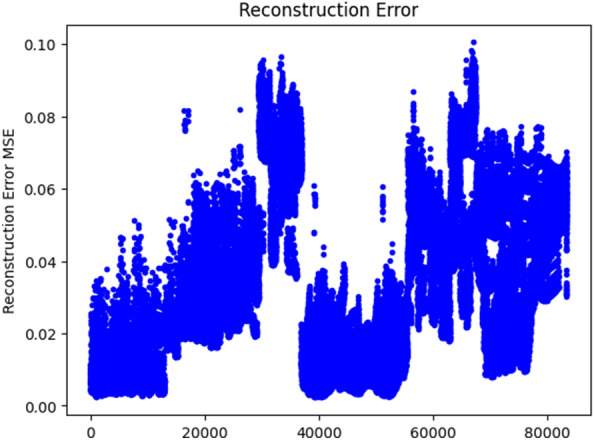
Traffic with Anomaly.

**Figure 4:**
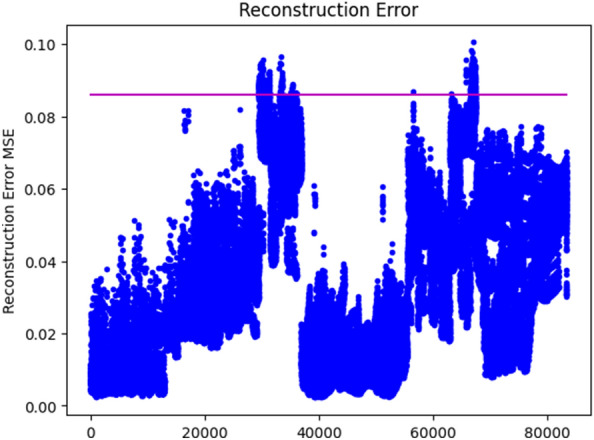
Anomaly removal threshold.

The plot of the reconstruction error on the train set is depicted in Fig. 3. The reconstruction MSE error, 0.091, is used as a threshold value to cluster anomaly and normal traffic flows. Values greater than 0.091 MSE error is grouped under useless for traffic Predicting as it is seen in Fig. 4.

**Figure 5:**
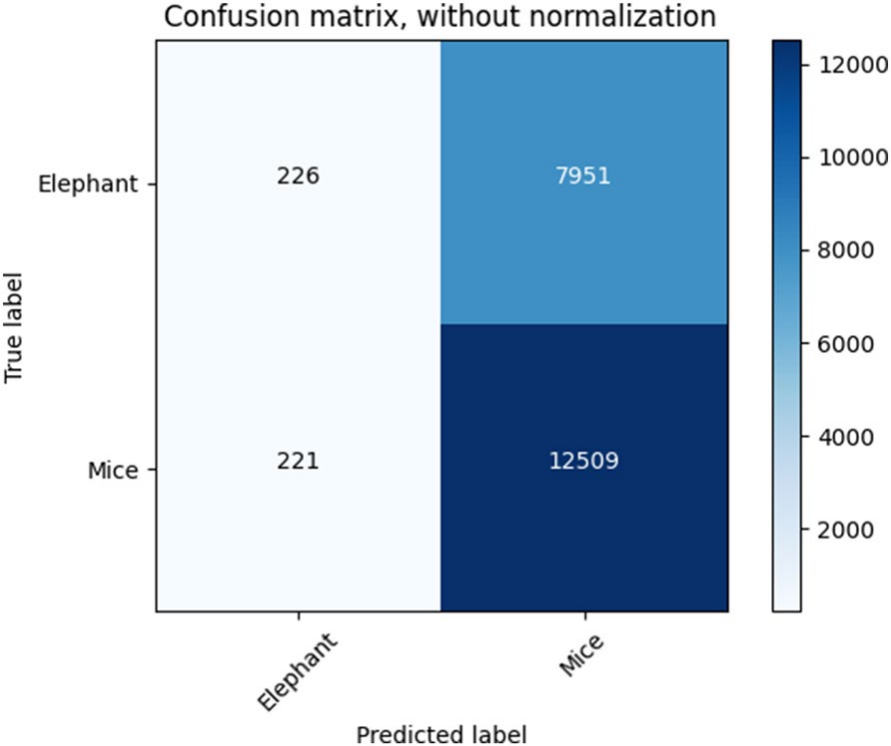
Confusion Matrix.

Data visualization is vital to understanding the relationship between mice and elephant traffic. Based on the threshold value, 0.091, the DNN auto-encoder model correctly predicted elephants as visualized in Fig. 5. True positive and true negative traffic are corrected and clustered to the intended cluster.

Confusion matrix is a prevalent measure used to understand classification models. It provides information about actual and predicted classes. It also shows the error matrix to describe the performance of a classification model on a set of test data.

226 instances were under elephant flow and these 226 instances are correctly predicted under the elephant category. 7951 were elephant flows in a true class assignment but these flows are expected under the mice category during prediction. 221 flows were categorized in elephant flows incorrectly, and now these 221 are predicted in the mice flows category. Whereas, 12,509 mice instances are correctly predicted and categorized into mice flow.

With an unsupervised model and threshold, new response elephant and mice labels were automatically given for each instance. We pretend that we don't know the label responses, and then the threshold for elephant determination can't be optimized for a confusion matrix.

Then, we accomplish supervised learning prediction with AutoML after setting essential hyperparameters for the performance in Table 3.

**Table 3:** Hyper-parameters.

Parameters	Setting
ax_runtime_secs	800
nfolds	5
include_algos	XGBoost, GBM, DRF
Seed	42

Prediction algorithms such as gradient boosting and random forest for classification involve a several hyper-parameters that must be set before running them^37^.

#### Traffic model prediction

Three AutoML algorithms XGBoost, GBM, and DRF, are used to develop the traffic classification model. We tested and compared the performance of these algorithms, XGBoost, GBM, and DRF algorithms. They performed 0.99%, 100% and 100% respectively in terms of training accuracy. XGBoost, GBM, and DRF yield MSE of 0.0000701, 0.0000895 and 0.0000055 respectively. The precision measure of these models is almost the same and losses are closer to zero assuring promising performance of the three built models. The training performance of the traffic Predicting model is presented in Table 4.

**Table 4:** Training accuracy, precision, loss.

	XGBoost	GBM	DRF
MSE	0.0000701	0.0000895	0.0000055
RMSE	0.008374838	0.009459834	0.002344221
LogLoss	0.00037684	0.000939903	0.0000318
Mean Per-Class Error	0.007575758	0	0
AUC	0.999999636	1	1
AUCPR	0.999999999	1	1
Gini	0.999999272	1	1

**Figure 6:**
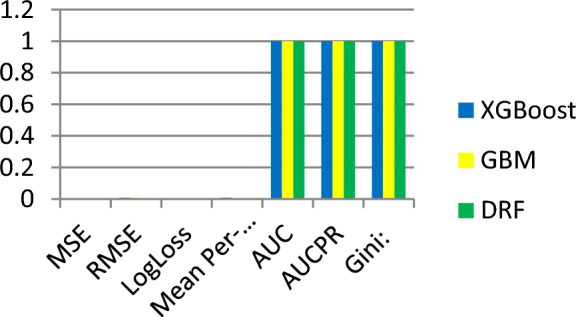
Training accuracy and MSE errors.

The training accuracy, precision, and loss of the three models is demonstrated in Fig. 6.

As the reported Validation accuracy is presented in Table 5, the validation accuracy of XGBoost, GBM, and DRF is 0.999667, 0.999973 and 1 under construction error of 0.0003952, 0.001697 and 0.00000408 respectively.

**Table 5:** Validation accuracy, precision, loss.

	XGBoost	GBM	DRF
MSE	0.000395276	0.001697421	0.00000408
RMSE	0.019881552	0.041199774	0.002019201
LogLoss	0.001507139	0.005966178	0.0000311
Mean Per-Class Error	0.117472258	0.034742372	0
AUC	0.999667073	0.999730117	1
AUCPR	0.999999471	0.99999403	1
Gini:	0.999334145	0.999460234	1

**Figure 7:**
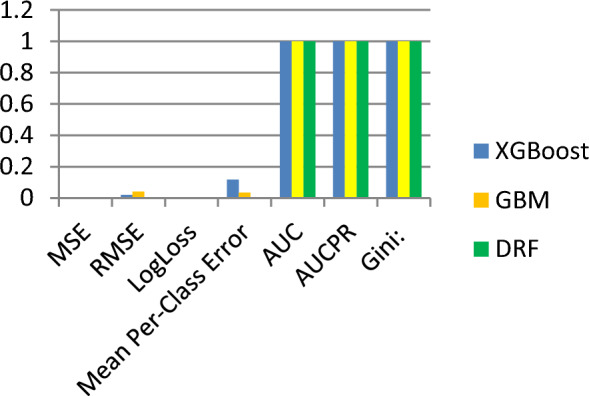
Validation accuracy and MSE errors.

The validation accuracy, precision, and loss of the three models is presented in Fig. 7.

The overall performance of the H_2_O clustering, Autoencoder and supervised prediction models performed very well in detecting elephant flows.

#### Result in comparison with related works

We compare the performance of our predicting model with some related works. For instance, Muna Al-Saadi et al.^2^ developed an elephant detection model using machine learning algorithms and achieved 88.2 accuracy. Silva, Marcus Vinicius Brito da et al.^4^, developed an elephant flow detection model that yields 90% accuracy with in less than 0.4 ms. Ali Malik and et al.^19^ also used deep learning and developed an elephant flow predicting model that performs 0.936% in average accuracy. On the other hand, our work employed XGBoost, GBM, and DRF algorithms for predicting elephant flows SDN networks. The validation accuracy of XGBoost, GBM, and DRF is 0.999667, 0.999973 and 1 respectively. Therefore, we conclude that these three model performances are much better than the related works in the paper^2^^,^^4, 19^. The XGBoost, GBM, and DRF models increase their accuracy results and recall by adjusting the threshold and detecting elephant flows more with the most minor reconstruction loss.

Pieter Barnard et al.^10^, explained the traffic identification model using SHAP in the local explanation technique. We compared the performance of our elephant predicting model using global explanation (SHAP) in addition to the local model.

To conclude, predicting elephant flow is the priority job of optimizing QoS in the SDN network.

**Experiment II:** Which factors or features have more importance for minimizing traffic congestion?

Good classification accuracy is the primary concern; however, identifying the attributes (or features) having the most significant classification power is attractive. Large datasets classification is highly dependent on feature selection. In addition to reducing the data dimensionality, selecting fewer and more representative attributes improves classification and yields better-preforming models^38^. Specifically, we used SHAP, an attribute and feature selection technique.

### Variable and feature importance

If variable importance is a top priority in a research analysis, it is better to consider training a Distributed Random Forest (DRF) model and comparing the generated variable importance^9^. While modeling the relationship between a dependent class variable and independent variables (inputs), Attribute importance is determined by the variables` contribution amount to some quantity of interest to the class as it is presented in Table 6.

**Table 6:** Variable importance.

Variable	Relative importance	Scaled importance	Percentage
Dst	738.7526855	1.0	0.2245657
Src	394.7110901	0.5342939	0.1199841
Switch	278.0320129	0.3763533	0.0845161
dur_nsec	273.2421265	0.3698696	0.0830600
Protocol	272.5742493	0.3689655	0.0828570
flows	261.1378479	0.3534848	0.0793806
Pktcount	195.3378754	0.2644158	0.0593787
Packetins	152.1798248	0.2059956	0.0462596
Bytecount	136.2605286	0.1844467	0.0414204
tot_kbps	119.2972717	0.1614847	0.0362639
pktrate	81.5488281	0.1103872	0.0247892
port_no	77.5333252	0.1049517	0.0235685
tot_dur	41.8599777	0.0566630	0.0127246
Dur	32.4385338	0.0439099	0.0098607
rx_bytes	31.0710964	0.0420589	0.0094450
Pktperflow	25.5084591	0.0345291	0.0077541
tx_kbps	20.8022137	0.0281586	0.0063234
rx_kbps	20.0869160	0.0271903	0.0061060
tx_bytes	13.6969090	0.0185406	0.0041636
Pairflow	6.3214526	0.0085569	0.0019216

The data value contribution also determines important of the features. Feature importance assigns a score to input features based on their usefulness in predicting a target attribute. The usefulness can be calculated based on statistical analysis, coefficient analysis and decision tree techniques^39^. The scores are used to better understand the dataset, a model and deduce number of features^40^. It can be used to improve a predictive model by deleting the lowest scores and keeping the highest scores. Deleting features is about dimensionality reduction to improve the model`s performance.

One of the techniques to select relevant features or reduce irrelevant feature is SHAP. SHAP helps investigate the importance of features in relative to target categorization^27^. For example, a host with source and destination address with 10.0.0.4 is a source of elephant flows as seen in Table 7. Therefore, src and dst addresses provide more heuristics to predict the occurrence of elephant flows whereas features such as port number values have lower score, which have less relevance for predicting elephant flows. Hence, a host with ip address 10.0.0.4 requires appropriate route reservation to render better QoS.

**Table 7:** Feature importance.

Feature	Relative importance	Scaled importance	Percentage
dst.10.0.0.4	1.0	1.0	0.0581569
src.10.0.0.4	0.7531736	0.7531736	0.0438022
src.10.0.0.12	0.6048512	0.6048512	0.0351763
dst.10.0.0.3	0.6038007	0.6038007	0.0351152
src.10.0.0.3	0.5998288	0.5998288	0.0348842
src.10.0.0.10	0.5928173	0.5928173	0.0344764
dst.10.0.0.5	0.4674018	0.4674018	0.0271826
src.10.0.0.1	0.4632869	0.4632869	0.0269433
Protocol.TCP	0.4463088	0.4463088	0.0259559
dst.10.0.0.12	0.4375176	0.4375176	0.0254447
Flows	0.1232236	0.1232236	0.0071663
tot_kbps	0.1067728	0.1067728	0.0062096
Switch	0.1007914	0.1007914	0.0058617
tx_bytes	0.1007665	0.1007665	0.0058603
rx_kbps	0.0977972	0.0977972	0.0056876
rx_bytes	0.0967681	0.0967681	0.0056277
port_no	0.0165301	0.0165301	0.0009613

As presented in Table 7, relative importance of the features is presented with their coefficients. It can be used directly to calculate scores which is not a must to be in between 0 and 1 value. Whereas scaled features scores are converted into fractions between 0 and 1.

The Shapley value is the average of all the marginal contributions to all possible input coalitions and features. SHAP values estimate the impact of a feature on predictions i.e. Feature importance estimate the impact of a feature on model fit. The goal of SHAP is to explain the prediction of an instance x by computing the contribution of each feature to the prediction. One of the visualizations you can produce is the force plot. Force plots identify features that contribute to the model’s prediction for a specific observation. SHAP is integrated into the tree-boosting frameworks xgboost, GBM and DRF. TreeSHAP is a variant of SHAP, including decision trees, random forests and gradient boosted trees. We trainee a random forest classifier to predict the occurrence of elephant flow for traffic management.

**Figure 8:**
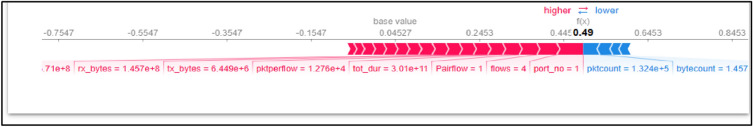
Traffic force plot.

**Figure 9:**
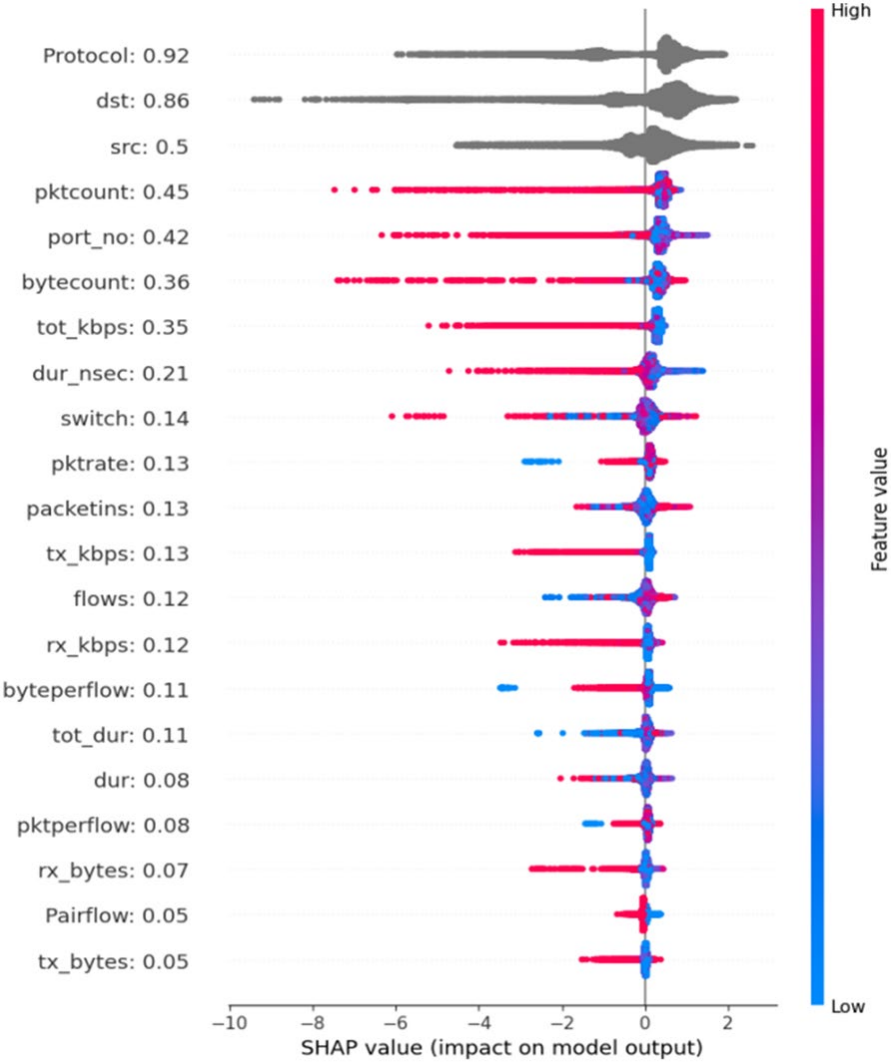
Feature Summary Plot.

In this work, we demonstrated the utility of SHAP to enhance the interpretation of QoS by network operators, experts and network administrators to prevent congestion in advance. The SHAP technique measures the importance of input traffic attributes on the Predicting model’s output^41^. We illustrate the value of the SHAP technique using Force plot and Summary plots in Figs. 8 and 9.

### Local interpretability using force plot

This work predicts whether a traffic flow is an elephant in the SDN network. The binary target was yes for elephant flows (flow = 1, or no; the flow may not bring network resources congestion (mice = 0). In Fig. 8, the bold 0.49 is the model’s score for this observation. Higher scores lead the model to predict 1 and lower scores lead the model to predict 0. The important features to predict traffic observation are shown in red and blue. Red represents features that pushed the model score higher, and blue represents features that pushed the score lower to prevent congestion. Features that had more of a normal impact on the score are located closer to the dividing boundary between red and blue, and the size of bar that represents the size of the impacts. As health network status, a congestion factor becomes at the baseline, 0.49 during data transmission i.e. underutilization and overutilization of resources could not be happened in this state.

Local interpretability allows analysing of the model classification for selected data points on SHAP force plots. SHAP values are associated with different “forces” that increase or decrease the model’s prediction. Each prediction starts from the base value of 0.49 which is given by the average of all probabilities for each traffic class present in the dataset if none of the input attributes are known^41^. In this study, the elephant and mice classes have a base-value probability of 0.49 and 0.51 respectively. It is important to note that the classification of sample #6402 as “elephant” was aided by low values of pairflows and flows, and high values of packet count and byte count. The top three attributes are tx_kbps, src and tot_kbps which are successfully identified using SHAP. Features with less and equal 0.49 including pairflow (1276e + 6), pktperflow (1276e + 4), paiarflow (1),flows (4).port_no (1) has less impact of driving elephants flows and the QoS is stabilized up to 0.49. On the other hand, pktcount (1.324e + 5) and bytecount (1.457) leads to elephant flows. Therefore, we need a mechanism to control a baseline (in our case, 0.49) feature setup to optimize QoS.

This plot can only be made for one traffic transmission observation. For this example, let us take the 6402nd record; port no 1, flow size with 4, pair flow = 1, total-duration 3.01e + 11 could not lead to elephant flows. On the other hand, pktcount (1.32e + 5) and byte count (1.457) leads to traffic congestion unless we push these traffic to lower sized traffic to better traffic control and prevent congestion. At the baseline, the average predicted probability is 0.49. This traffic flow has high predicted congestion if it is greater than 0.49. Therefore, the increasing effects from attributes including Port no 1, flow size with 4, pair flow = 1, total duration 0.01e + 11 is recommended to wisely use bandwidth. The decreasing effects of pktcount and bytecount from 1.32e + 5 and 1.457, prevents overutilization of bandwidth and optimize QoS.

### Global interpretability using summary plot

SHAP is based on the effect of feature attributions. The feature importance plot is useful, but contains no information beyond the importance^42^. Look at the summary plot in Fig. 9 for a more informative plot.

**Figure 10:**
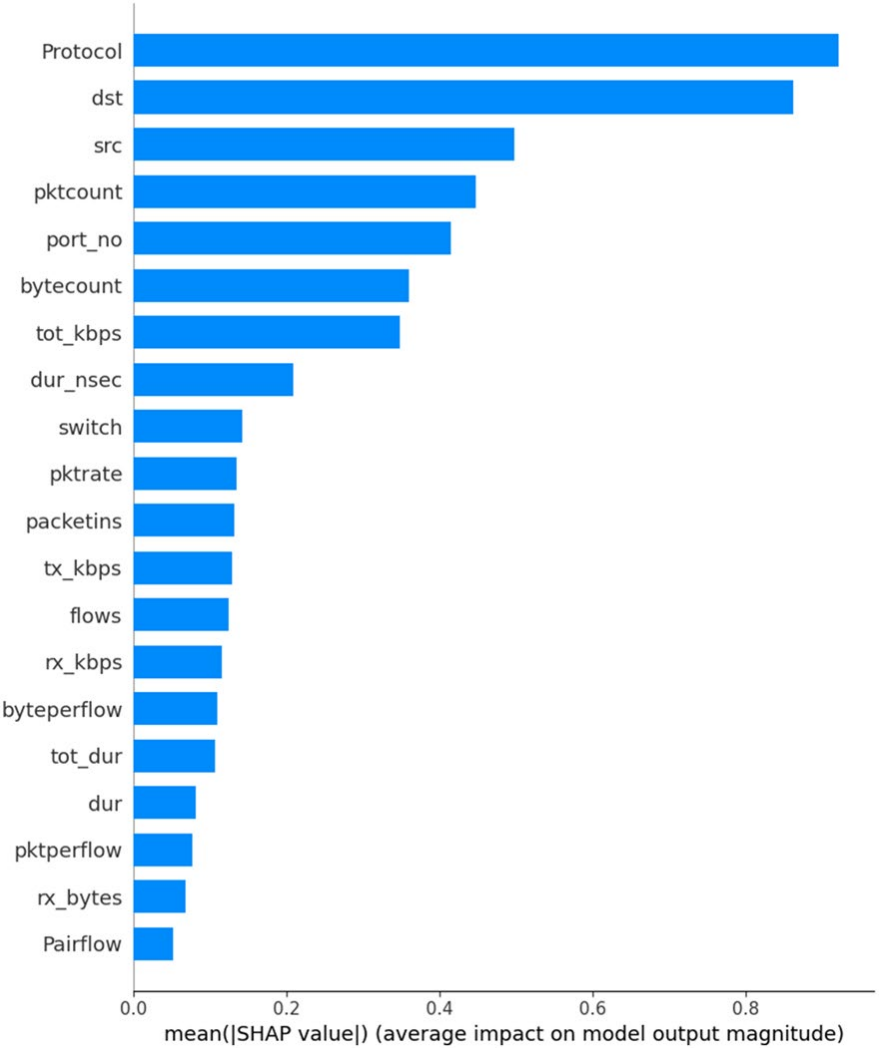
SHAP feature importance.

The summary plot combines feature importance with feature effects. Each point on the summary plot is a Shapley value for a feature and an instance^27^. The position on the y-axis is y-axis direction, so we understand the distribution of the shapely values per feature. The color represents the value of the feature from low to high. Overlapping points are jittered in the y-axis direction, so we understand the distribution of the Shapley values per feature are ordered according to their importance. SHAP positive values increase the probability of elephant flows, and are associated with protocol values: source address, destination address, packet account and byte count. Features with large absolute Shapley values are important. Since we want global importance as it is seen in Fig. 10.

SHAP feature importance is measured as the mean absolute Shapley values and hence, the predicted absolute elephant probability on average by 92% percentage points (0.92 on the x-axis). From 21 candidate traffic attributes, the highest contribution is associated with the protocol, followed by source address, destination address, and packet count when predicting elephant and mice flows.

## Conclusion and recommendation

### Conclusion

Identifying elephant flows is very important to take pre-action before network congestion happens. To prevent congestion in advance and optimize QoS, we used the H2O deep learning algorithm for traffic clustering for labeling traffic instances automatically. Then, we also detected anomalies from labeled datasets and remove them using deep Autoencoder before predicting model development.

The Autoencoder sets a threshold to identify anomaly flows among normal elephant and mice flows.

After labeling the automatic class using H2O and cleaning the dataset from anomalies using deep authoencoder, we developed elephant prediction models using autoML algorithms including XGBoost, GBM, and DRF algorithms.

Model performance results show that the algorithms outperform during learning and validation on the SDN dataset. Hence, the performance of the models was very promising to provide good QoS provision.

We also explained the balck-box nature of deep learning models explicitly using SHAP to show the importance of features and attribute analysis to prevent negative effects of elephant flows and maximize QoS. However, the limitation of our model is that, predicting model integration was not experimented with in the SDN testbed environment.

### Future works

The automatic traffic prediction is run and tested SDN Datasets using Google Colab laboratory. For future work, the predicting model will be integrated ino SDN controller, RYU and the actual performance will be measured in terms of throughput. Furthermore, it was better to test the models in a testbed of real SDN controllers and openswiches. Therefore, additional experimentation concerning integrating these models and evaluating their performance regarding the load, speed, and accuracy is the recommended point in both SDN simulation and physical testbed integration.

## Data Availability

The SDN datasets are available at: https://data.mendeley.com/datasets/jxpfjc64kr/1.

## Abbreviations

XAI: Explainable Artificial Intelligence
DPI: Deep packet inspection
ISPs: Internet service providers
GBM: Gradient boosting machine
GDF: Gradient distributed forest
UDP: User datagram protocol
SHAP: Shapley additive explanations
QoS: Quality of service
TCP: Transmission control protocol
VoIP: Voice over internet protocol
ML: Machine learning
